# Use of an on/off tetracycline riboswitch to control protein production in *Komagataella phaffii*

**DOI:** 10.1186/s13568-023-01637-5

**Published:** 2023-11-21

**Authors:** Igor Patrick Vasconcelos Vieira, Felipe Seixas Arreguy Pimentel, Cintia Marques Coelho, Janice Lisboa De Marco, Lidia Maria Pepe de Moraes, Fernando Araripe Gonçalves Torres

**Affiliations:** 1 Laboratório de Biologia Molecular, Instituto de Ciências Biológicas, Universidade de Brasília, Distrito Federal, Brazil; 2 Laboratório de Biologia Sintética, Instituto de Ciências Biológicas, Universidade de Brasília, Distrito Federal, Brazil; 3 Laboratório de Biotecnologia de Leveduras, Instituto de Bioquímica Médica, Universidade Federal do Rio de Janeiro, Rio de Janeiro, Brazil

**Keywords:** *Komagataella phaffii*, Riboswitch, Tetracycline, Aptamer, Translational control of gene expression

## Abstract

The methylotrophic yeast *Komagataella phaffii* is one of the most important microbial platforms to produce recombinant proteins. Despite its importance in the context of industrial biotechnology, the use of synthetic biology approaches in *K. phaffii* is hampered by the fact that few genetic tools are available for precise control of gene expression in this system. In this work, we used an RNA aptamer activated by tetracycline to modulate protein production at the translational level. Using *lacZ* as gene reporter, we have demonstrated significant reduction of the heterologous protein upon addition of tetracycline. Furthermore, this genetic control device was applied for the control of Ku70p. This protein is involved in non-homologous recombination and the control of its production paves the way for the development of strains exhibiting higher rates of homologous recombination.

**Supplementary Information:**

The online version contains supplementary material available at 10.1186/s13568-023-01637-5.

## Introduction

*Komagataella phaffii* (formerly *Pichia pastoris*) is a methylotrophic yeast which was initially considered as source of single cell protein in the 1970’s. Today, it has emerged as a powerful chassis organism to produce recombinant proteins (Li et al. 2007). Due to its ability to perform post-translational modifications and to secrete a plethora of proteins and peptides, it has gained much attention in the context of industrial biotechnology (Baghban et al. 2019). Furthermore, entire metabolic pathways, such as those related to the synthesis of riboflavin (also known as vitamin B2), lycopene and β-carotene (precursors of vitamin A), were successfully introduced in *K. phaffii* thus showing its high applicability to produce metabolites of biotechnological interest (Araya-Garay et al. 2012; He et al. 2006; Marx et al. 2008). However, the assembly and precise integration of multiple fragments into the genome is somehow hampered by the fact that *K. phaffii* is not as efficient in integrating DNA fragments through homologous recombination (HR) as *Saccharomyces cerevisiae* (Camattari et al. 2016; Yu et al. 2012).

The molecular genetics in *K. phaffii* is essentially based on the molecular tools developed with great success for *S. cerevisiae* (Gomes et al. 2018). The most widely used approach to genetically modify strains of *K. phaffii* is by using integrative vectors which offer greater mitotic stability and therefore are considered well suited for recombinant protein production. Episomal vectors are less common and still require the isolation of new replication origins to offer better alternatives for the genetic manipulation of *K. phaffii* (Camattari et al. 2016). Commonly used promoters include P_GAP_ (glyceraldehyde 3-phosphate dehydrogenase) and P_AOX1_ (alcohol oxidase I), for constitutive and inducible expression, respectively (Vogl and Glieder 2013). P_AOX1_ is tightly controlled by methanol, but the toxicity and flammability of this inducer is a major problem in large scale processes (Shen et al. 2016). Other promoters have been tested but are not largely used due to problems related to weak expression, restricted conditions for induction and transcription leak in the absence of stimulus (Tschopp et al. 1987; Shen et al. 1998; Stadlmayr et al. 2010; Prielhofer et al. 2013; Ahmad et al. 2014).

The correct balance between promoter strength and expression control is essential for heterologous protein production. Weak promoters will not produce enough transcripts and therefore will reduce production yield. On the other hand, excessively strong promoters could lead to energetic burden, overloading the cell metabolism, slowing cell growth, increasing misfolded proteins and consequently reducing production yield (Gomes et al. 2018). To address this issue, the use of small ligand-binding aptamers is a viable alternative for controlling mRNA translation (Hanson et al. 2003). These sequences can be easily introduced by PCR adjacent to the ribosome binding site or to the start codon of an open-reading frame (ORF). As the riboswitch is transcribed, the ligand can bind to it with high affinity and specificity thus resulting in a unique 3D structure which may block protein translation (Hanson et al. 2003; Redden et al. 2015). Although the application of aptamers for the control of gene expression is virtually suitable for any promoter it has not yet been tested in *K. phaffii*. As a proof of concept, in this work we have analyzed the application of a tetracycline-binding aptamer as an “on–off switch” to control protein production in *K. phaffii*.

## Materials and methods

### Media and strains

*Escherichia coli* XL-10 Gold (Agilent Technologies) was used for construction and propagation of vectors used in this work. *K. phaffii* X-33 strain (Invitrogen) was the selected strain as control and background strain for genetic manipulations. Bacterial strains were grown using LB broth (0.5% Yeast Extract, 1% Tryptone and 1% NaCl, pH 7.2) at 37 °C under 150 rpm agitation. LB plates were poured using 1.5% agar supplemented with 25 µg/mL zeocin for selective pressure and maintenance of plasmids pPICH-lacZ-3tc and pPICH-ku70-3tc. Yeast strains were grown on YPD medium (1% Yeast Extract, 2% Peptone and 2% Glucose) under 200 rpm agitation at 30 °C. YPD plates were poured with 2% agar and supplemented with 100 µg/mL zeocin whenever necessary. The blue and white colony assay was performed on YPD plates containing zeocin and 2% top agar X-Gal. Tetracycline was added to the medium at different concentrations ranging from 5 to 100 µM.

### lacZ and ku70p expression vector construction

The backbone vector used for the cloning of the *lacZ* reporter gene was pPICHOLI (Mobitec). The vector was digested with BamHI and NotI restriction enzymes (New England Biolabs) to release the *AOX1* promoter, ORF and terminator sequences. The resulting linearized plasmid was named pPICH. Then, *lacZ* reporter gene was amplified by PCR from pLG∆178 (Guarente and Mason 1983) using Phusion DNA polymerase (New England Biolabs) under the following conditions: initial denaturation at 98 °C for 1 min followed by 40 cycles of 98 °C for 30 s, 60 °C for 30 s and 72 °C for 2 min; final extension at 72 °C for 5 min. Reaction was set according to the manufacturer’s instructions manual and primers lacZ-Fw and lacZ-Rv (see Additional file 1: Table S1) were used at a final concentration of 0.25 µM. The *lacZ* fragment was cloned into pPICH vector using the NEBuilder HiFi DNA assembly kit (New England Biolabs) on a proportion 1:2 vector-insert with 50 ng of total DNA. The reaction was incubated at 50 °C for 60 min and subsequently used to transform chemically competent *E. coli* XL-10 Gold. Transformants were selected on LB plates containing zeocin and correct insertion was further confirmed by PCR and restriction analysis. The resulting vector was named pPICH-lacZ. The *KU70* gene (GenBank accession number CP014717.1) was amplified by PCR from genomic DNA extracted from *K. phaffii* GS115 (Invitrogen). Primers ku70-Fw and Ku70-Rv were used for amplification and PCR conditions were similar to those used for *lacZ* amplification. The resulting vector was named pPICH-ku70. HA-tag was cloned into pPICH-ku70 via PCR with the primers described in Additional file 1: Table S1. The tetracycline aptamer was synthesized by GenOne and clone into pUC19 plasmid. The sequence was amplified by PCR, purified, digested with SphI and NdeI (New England Biolabs) and cloned into pPICH-lacZ and pPICH-ku70 plasmids. *K. phaffii* transformants are mentioned as TRS (tetracycline-responsive strain) Blue when transformed with pPICH-lacZ-3tc and TRS ku70 when transformed with pPICH-ku70-3tc. Both plasmids contain three sequences of the tetracycline riboswitch controlling the ORFs. Plasmid maps used for negative control without the tetracycline riboswitch are shown in Additional file 1: Fig. S1. Sequences of all plasmids used in this work are available in Additional files 3 and 4.

### β-Galactosidase activity assay

Cells were grown overnight at 30 °C and 200 rpm agitation on YPD supplemented with zeocin and tetracycline. Then, cells were harvested by centrifugation at 3000×*g* for 5 min. An aliquot of these cells was reinoculated in fresh medium for a final concentration of 0.1 OD_600nm_. Cells were grown up to ~ 0.7–0.8 OD_600nm_ and a total of 3 OD_600nm_ were collected by centrifugation. The pellet was resuspended in 1 mL Z buffer (60 mM Na_2_HPO_4_, 10 mM KCl and 1 mM MgSO_4_). Cell permeabilization was performed by adding 25 µL of 0.1% SDS and 50 µL chloroform followed by 3 cycles of strong agitation for 30 s, incubation at 30 °C for 15 min and 5 min at − 80 °C. Then, reaction was started by adding 200 µL of 4 mg/mL ONPG solution at 30 °C. The reaction was stopped after 5 min when solution turned yellow by addition of 500 µL of 1 M NaCO_3_. Measurement of 600 nm absorbance was performed immediately and 420 nm absorbance was done after separation of cell debris by centrifugation. β-Galactosidase activity was calculated by the following formula:

$$Miller\;units={1000} \times \left(\frac{OD420{\text{nm}}}{OD600{\text{nm}}* V*T}\right),$$

where T is the reaction time (min) and V is the volume (mL) collected from culture. The data used for calculations in this work are provided in the Additional file 2.

### Statistical analysis

All samples were analyzed using GraphPad Prism software. Unpaired t-Test was calculated from three independent experiments with triplicates of every sample each time. Figures are representative of the results and show mean values and standard deviation.

### Protein extraction

One isolated colony was inoculated in liquid YPD media containing zeocin (100 µg/mL). The culture was kept at 30 °C under 200 rpm agitation for 24 h. Cells were collected by centrifugation at 2000×*g* for 5 min and washed twice with sterile water. Then, a new batch was prepared by adding cells to a final concentration of 0.1 OD_600nm_ on fresh medium at 30 °C and 200 rpm agitation until cell concentration was ~ 1.0 OD_600nm_. The culture was centrifuged for 10 min at 5000×*g* at 4 °C and the pellet was resuspended in 450 µL of ice-cold extraction buffer (50 mM Tris-HCl, 1.25% Tween 20 [pH 8.8]) and 200 μm glass beads were added. The extraction buffer was supplemented with a protease inhibitor cocktail (cOmplete Protease Inhibitor, Roche) prior to cell lysis. Protein extraction was performed by 10 cycles of 30 s under intense agitation with Precellys Lysing kit (Bertin) and 1 min of ice-cold water bath. The extract was collected with the aid of a pipette and placed in another ice-cold tube. Extraction buffer (200 µL) was added to the glass beads and vortexed to retrieve the remaining extract trapped and mixed with the volume obtained before. The whole protein extract was centrifuged at 18,000×*g* for 30 min at 4 °C and the supernatant containing soluble proteins was collected and stored at − 20 °C until use.

### Protein concentration determination

Protein concentration was determined by using BioRad Protein Assay Dye Reagent. The standard curve was made using BSA accordingly to the concentration of the sample.

### Polyacrylamide gel electrophoresis (PAGE) and Western blotting

Protein extracts were initially analyzed in SDS-PAGE (5% stacking, 12% resolving gel). Samples were boiled for 5 min in loading dye containing 100 mM Tris-HCl pH 6.8, 8 M urea, 20% glycerol, 4% SDS, 0.2% bromophenol blue and 100 mM β-mercaptoethanol prior to application. Electrophoresis was performed at room temperature at a constant current of 0.05 A. Gel content was evaluated with Coomassie Brilliant Blue G-250 staining (Merck). Every gel was prepared in duplicates as one was used for Western blotting. Protein transfer to nitrocellulose membranes (Merck Millipore) were performed at a constant current 0.15 A at room temperature for 60 min. The quality of the transfer was evaluated by dying the membrane with Ponceau S. Membrane was blocked for 1 h using 5% skim milk solution reconstituted in TBST buffer. Then, the membrane was incubated with a 1 µg/mL solution of monoclonal rabbit anti-HA antibody (SAB5600193, Sigma Aldrich) in TBST buffer supplemented with 3% BSA for 1 h at room temperature. Secondary anti-rabbit antibody conjugated with peroxidase was used for revelation after 1 h of incubation at room temperature. Revelation was performed by incubating the membrane with revealing buffer (Abcam) for 2 min in a dark chamber. Chemiluminescence was obtained on semi-automatic capture mode after 1 min of exposure on the Imager 680 (Amersham). The membrane was washed with TBST three times for 5 min each prior to every incubation step (blocking, primary and secondary antibody and revelation). Measurement of band densitometry was performed on ImageJ 1.54 using area under the peak method. Background was excluded from measurement. No editing was performed in the images presented. Contrast and brightness were adjusted for better visualization of the data. Ku70p estimated size: ~ 60 kDa.

## Results

**Fig. 1:**
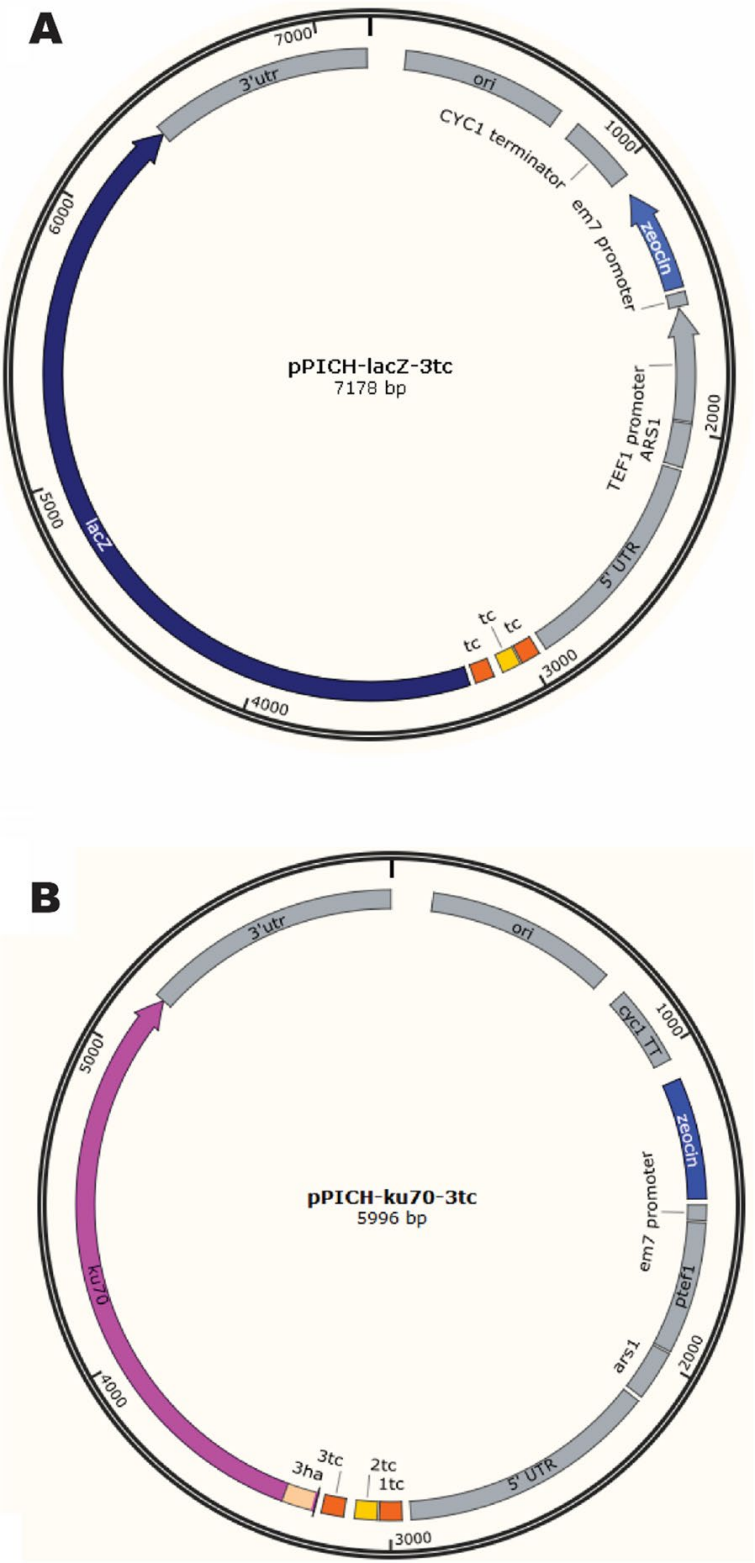
Map of the plasmids containing the tetracycline aptamer (tc) controlling protein production. **A** pPICH-lacZ-3tc episomal vector containing *lacZ* under control of the tc aptamer. **B** pPICH-ku70-3tc episomal vector containing ku70 under control of the tc aptamer. All sequences were amplified by PCR and cloned into pPICH vector by homologous recombination or via PCR

**Fig. 2:**
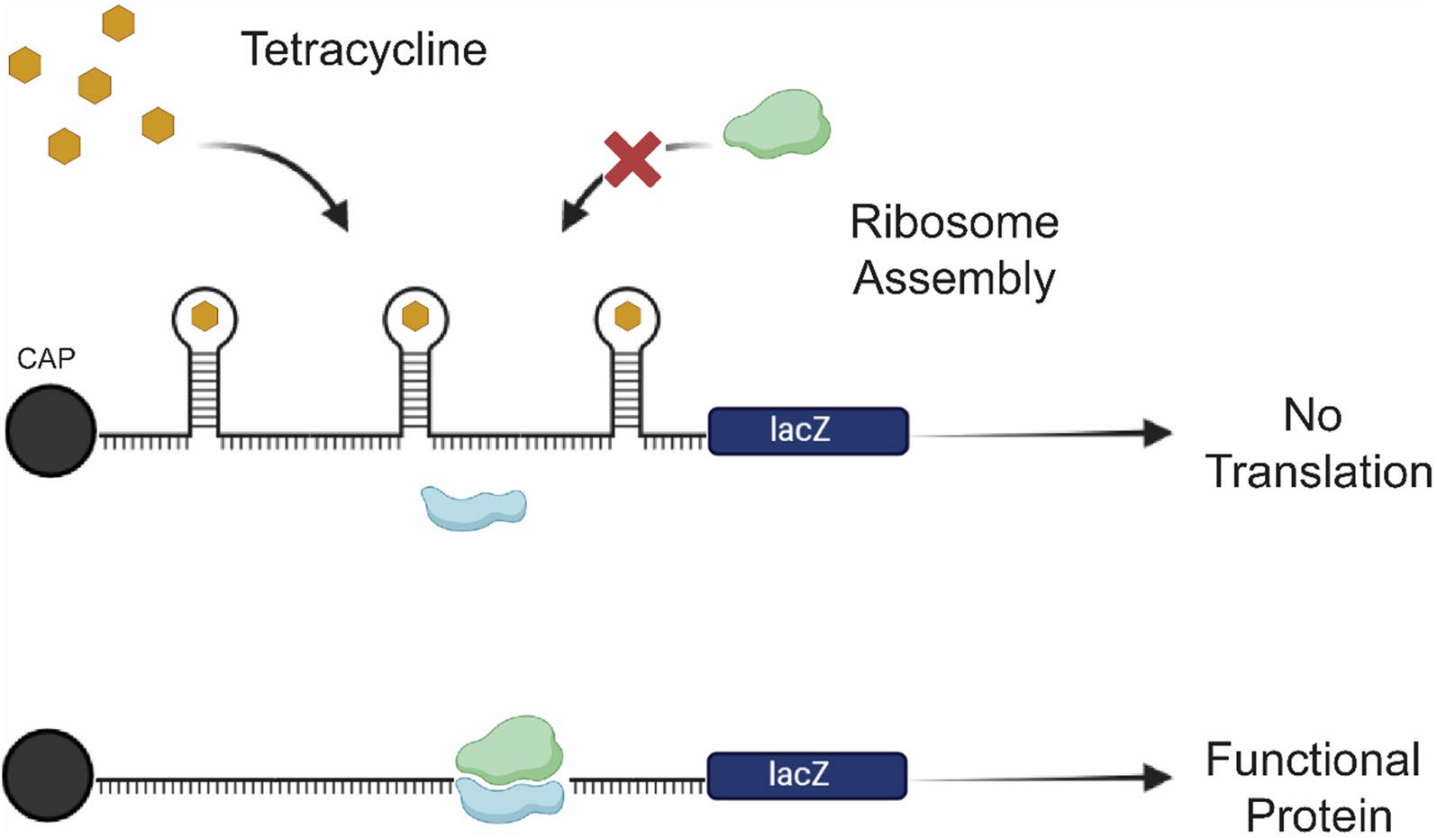
Description of the on-off tetracycline regulation system on mRNA translation. On the absence of tetracycline, the ribosome binds to the mRNA and translates to a protein. When tetracycline is present, the aptamer folds itself into a tridimensional structure that prevents ribosome assembly thus reducing protein production

In order to develop an expression system regulated by the tetracycline-regulated aptamer, we used the episomal vector pPICH as a backbone (Fig. 1A), which ensures high stability and multiple copies. The tetracycline aptamer and *lacZ* reporter gene were amplified by PCR and cloned into linearized pPICH. The resulting vector named pPICH-lacZ-3tc contains three aptamer sequences *in tandem* to allow tetracycline binding. Furthermore, to control expression of *lacZ*, the aptamers were placed at position − 21 bp from the start codon. Therefore, in the presence of tetracycline, the mRNA would fold and create a 3D structure that prevents protein translation by the ribosome as shown on Fig. 2.

**Fig. 3:**
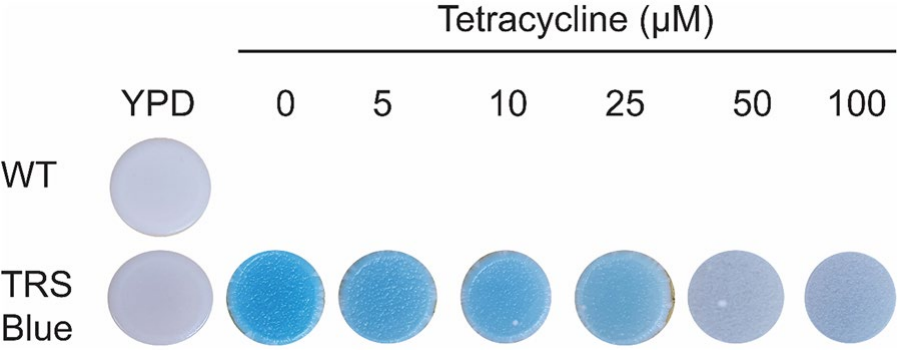
The tetracycline aptamer hampers *lacZ* production in *K. phaffii*. Cells were incubated on YPD plates containing X-GAL for 3 days at 30 °C and 4 days at 4 °C to enhance blue color intensity. Increasing concentrations of tetracycline show a dose-dependent inhibition response, preventing X-Gal metabolism and decreasing the blue color intensity in higher concentrations. Figure is representative of three independent experiments with triplicates of each spot on every plate

To ensure its proper function, the riboswitch system was tested in the presence or absence of tetracycline. For that, *K. phaffii* clones containing the riboswitch system were spotted on a YPD plate containing top agar X-Gal so that those exhibiting β-galactosidase activity would present blue spots. As shown on Fig. 3, colonies that were exposed to tetracycline concentrations of 50 µM or higher exhibited a reduction on the intensity of the blue color, a result consistent with a functional aptamer, as previously observed by Hanson et al. (2003).

**Fig. 4:**
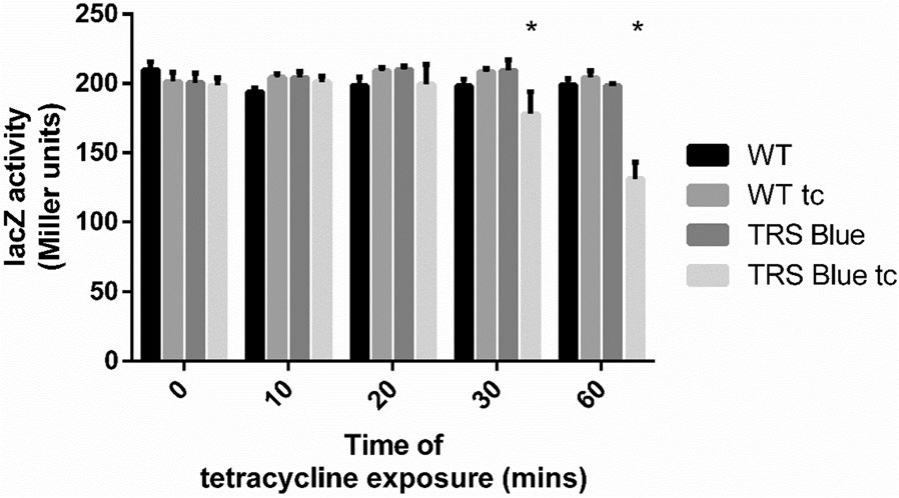
β-Galactosidase activity is reduced after exposure to tetracycline. The TRS Blue strain has shown to decrease protein production after 30 min of exposure in liquid media (n = 3). *Unpaired t-test shows that TRS Blue tc samples are significantly different from the other groups tested at a 95% confidence interval

As the plate assay with X-Gal requires longer incubation times, it was necessary to evaluate cell response in a shorter window time. We assessed β-galactosidase activity from 10 to 60 min of exposure time and 100 µM tetracycline with the ONPG assay (Fig. 4). The results show a reduction of ~ 34% in enzyme activity after 60 min exposure.

**Fig. 5:**
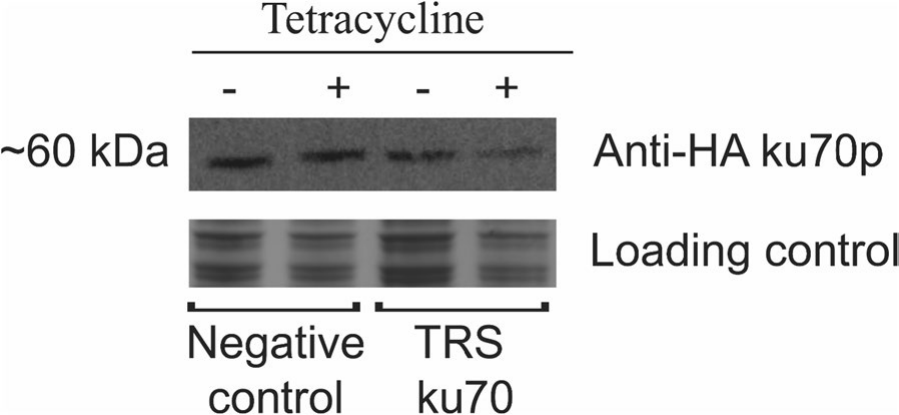
Reduction of Ku70p expression by influence of tetracycline. TRS ku70 strain exposure to 250 µM tetracycline for 8 h has shown approximately 40% of reduction in protein translation. Ku70p calculated expected size: ~ 60 kDa

Since the β-galactosidase assay showed the functionality of the tetracycline-aptamer system in *K. phaffii*, we decided to further investigate if another protein would have similar results. The protein of choice was ku70p as it has been reported to show an important role on double-strand DNA damage repair system. *KU70* mutants do not display a visual phenotype, we fused an HA-tag to the N-terminal of ku70p to detect protein production by Western blotting. The tagged gene was cloned into pPICH in a similar manner as *lacZ*, creating the plasmid pPICH-ku70-3tc (Fig. 1B), responsive to tetracycline. The system was re-tested after the addition of 250 µM tetracycline to the media and 8 h of exposure. In this case, results show that ku70p concentration was reduced by ~ 40% (Fig. 5). Therefore, the tetracycline riboswitch was activated by the presence of tetracycline and has successfully controlled the translation of both *lacZ* and ku70p.

## Discussion

The use of the tetracycline riboswitch is a cost-effective strategy for controlling expression of virtually any protein. As the system allows precise control at the translational level, genes bearing both strong and weak promoters may be controlled in the same manner. This implies that less genomic manipulations are necessary. Also, by increasing the number of repetitive tetracycline binding sites, a more restrictive control of protein translation may be achieved regardless of the strength of the promoter (Hanson et al. 2003; Suess et al. 2003; Berens et al. 2015; Ruscito and DeRosa 2016).

Considering the observations made with *lacZ*, if the white colonies resulting from the exposure to tetracycline are removed from the presence of the antibiotic, translation is resumed and leads to the development of the intense blue color rapidly, since mRNA expression is not altered. The ability to reverse the output of the genetic control is a key feature of the tetracycline riboswitch. This represents a quick response of the aptamer-based system to stimulus. However, β-galactosidase is known to have a short life span in the cell and other proteins with longer half-life might not reduce their activity or concentration as fast as β-galactosidase. Therefore, a case-by-case analysis should be observed for every protein tested in which exposure time and tetracycline concentration may play critical roles.

As the β-galactosidase assay showed the functionality of the tetracycline-aptamer, ku70p was the next protein of choice to show the applicability of the riboswitch system. This protein has been reported to show an important role on the double-strand DNA damage repair system. Although ku70p’s map of interactions and pathways have not yet been fully stablished, it is well known that it binds to ku80p in a dimer and recruits other proteins involved in the repairing machinery of the DNA structure (Schwartz et al. 2017; Weninger et al. 2016). It also has been described by Näätsaari et al. (2012) that the use of longer homologous sequences for integration and the deletion of *KU70* have shown to reduce non-homologous end-joining (NHEJ) while simultaneously increasing HR. As the NHEJ system causes non-specific integration of exogenous DNA, it leads to reduction of transformation efficiency. Consequently, the selection of correct transformants after genetic manipulation in *K. phaffii is* usually a very laborious effort since this yeast has a very active NHEJ system. Therefore, strains that have a diminished NHEJ and increased HR rate are highly desired. However, the prolonged absence of ku70p in *K. phaffii* has shown to increase non-canonical events of chromosome translocation leading to significant changes in physiological responses and DNA structure (Schwarzhans et al. 2016). In the light of these observations, it is desirable to control the expression of ku70p in a reversible manner in order to avoid these effects, hence the choice for a riboswitch.

As HR depends on multiple variables to determine its efficiency such as length of the recombination sequences, loci of integration and DNA quantity, further studies are required to assess the significance of this result in the improvement of HR in *K. phaffii*.

In conclusion, this work has shown that the use of RNA aptamers for controlling protein production in *K. phaffii* is feasible and should represent a significant contribution to the genetic toolbox of this important chassis organism.

### Supplementary Information

**Additional file 1: Table S1.** PCR primers used in this work. **Figure S1.** Plasmids used in X-33 strain to create negative control strains. The absence of the riboswitch makes the strain irresponsible to tetracycline. **Figure S2.** Whole SDS Page gel used for western blotting. Excerpt marked in red corresponds to expected ku70p band size. No editing was performed in this image. Lane 1: Protein molecular weight ladder. Lanes 2 and 3: Loading control. Lane 4: 50 µg Negative control (no riboswitch) and no tetracycline. Lane 5: 50 µg Negative control (no riboswitch) 250 µM tetracycline. Lane 6: 50 µg TRS ku70 no tetracycline. Lane 7: 50 µg TRS ku70 250 µM tetracycline. **Figure S3.** Whole image of western blotting membrane used in figure 5. No editing was performed in this image. Lane 1: Protein molecular weight ladder. Lanes 2 and 3: Loading control. Lane 4: 50 µg Negative control (no riboswitch) and no tetracycline. Lane 5: 50 µg Negative control (no riboswitch) 250µM tetracycline. Lane 6: 50 µg TRS ku70 no tetracycline. Lane 7: 50 µg TRS ku70 250 µM tetracycline.

**Additional file 2:** Spreadsheet containing the data used for calculation of β-Galactosidase activity and Western blotting densitometry. Results are presented in Figures 4 and 5. Descriptive statistics are shown in the file.

**Additional file 3:** Map of the plasmid pPICH-lac-3tc and its sequence.

**Additional file 4:** Map of the plasmid pPICH-ku70-3tc and its sequence.

## Data Availability

All data is available and free of charges in the additional files. Availability of materials and shipment must be addressed individually with the corresponding author.

## Abbreviations

BSA: Bovine serum albumin
GFP: Green fluorescent protein
HA-tag: Human influenza hemagglutinin tag
HR: Homologous recombination
mRNA: Messenger ribonucleotide acid
NHEJ: Non-homologous end joint
OD_600nm_: Optical density at 600 nm
ONPG: 2-Nitrophenyl β-d-galactopyranoside
PCR: Polymerase chain reaction
RBS: Ribosome binding site
TRS: Tetracycline-responsive strain
