# Supplementary material for: Use of an on/off tetracycline riboswitch to control protein production in Komagataella phaffii
Source: AMB Express. 2023 Nov 21;13:131. doi: 10.1186/s13568-023-01637-5 (PMC10663417; doi:10.1186/s13568-023-01637-5)
Supplement: Supplementary file 1 — Additional file 1: Table S1. PCR primers used in this work. Figure S1. Plasmids used in X-33 strain to create negative control strains. The absence of the riboswitch makes the strain irresponsible to tetracycline. Figure S2. Whole SDS Page gel used for western blotting. Excerpt marked in red corresponds to expected ku70p band size. No editing was performed in this image. Lane 1: Protein molecular weight ladder. Lanes 2 and 3: Loading control. Lane 4: 50 µg Negative control (no riboswitch) and no tetracycline. Lane 5: 50 µg Negative control (no riboswitch) 250 µM tetracycline. Lane 6: 50 µg TRS ku70 no tetracycline. Lane 7: 50 µg TRS ku70 250 µM tetracycline. Figure S3. Whole image of western blotting membrane used in figure 5. No editing was performed in this image. Lane 1: Protein molecular weight ladder. Lanes 2 and 3: Loading control. Lane 4: 50 µg Negative control (no riboswitch) and no tetracycline. Lane 5: 50 µg Negative control (no riboswitch) 250µM tetracycline. Lane 6: 50 µg TRS ku70 no tetracycline. Lane 7: 50 µg TRS ku70 250 µM tetracycline. [file 13568_2023_1637_MOESM1_ESM.docx]

| **Name** | **Sequence** |
| --- | --- |
| LacZ-Fw | TCGAGAACATATGGGGGATCCATGACCGGATCCGGAG |
| LacZ-Rv | ACGTTGCGAGGAAAAAGTAAGGAACACGAATTATTTTTGACAC |
| Ku70-Fw | TGGTCTGGTGTCAAAAATAATTCGTGTTCCTTACTTTTTCCTCG |
| Ku70-Rv | CTCCGGATCCGGTCATGGATCCCCCATATGTTCTCGA |
| 3HA-Fw | ATGTACCCATACGATGTTCCAGATTACGCTTACCCATACGATGTTCCAGATTACGCTTACCC ATACGATGTTCCAGATTACGCTAGTGTTGTCAGCAAGCAAAGTGTTGTCAGCAAGCAATAC |
| 3HA-Rv | AGTAAACCCGCTTGATCTCCTTATGTCTAGAAATTCGTTGGCGGATCTCCTCAAT |
| 3tcapt-Fw | TATAGAGGAAACGGGGTTACGTGCAAGCGCGCATGCCTGAGCTTTGAGGG |
| 3tcapt-Rv | AGCGTAATCTGGAACATCGTATGGGTACATGGATCCCCCATATGTTCTCGA |

**Table S1: PCR Primers used in this work.**


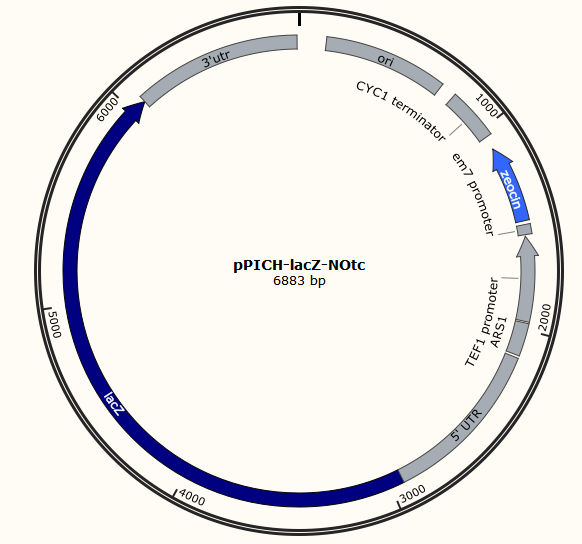


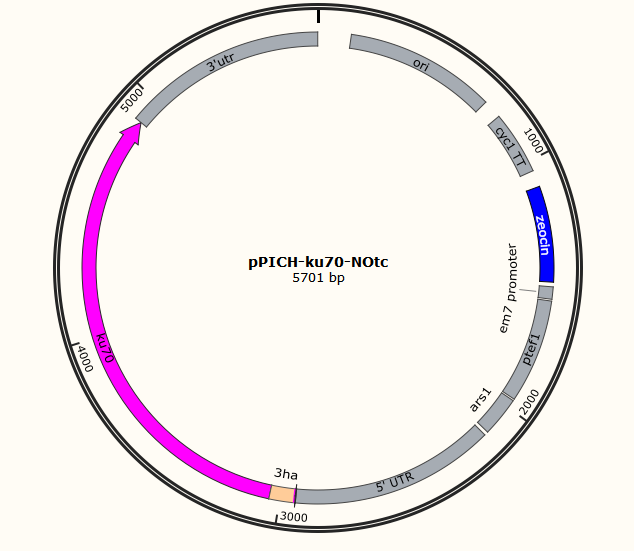


**Figure S1: Plasmids used in X-33 strain to create negative control strains.** The absence of the riboswitch makes the strain irresponsible to tetracycline.


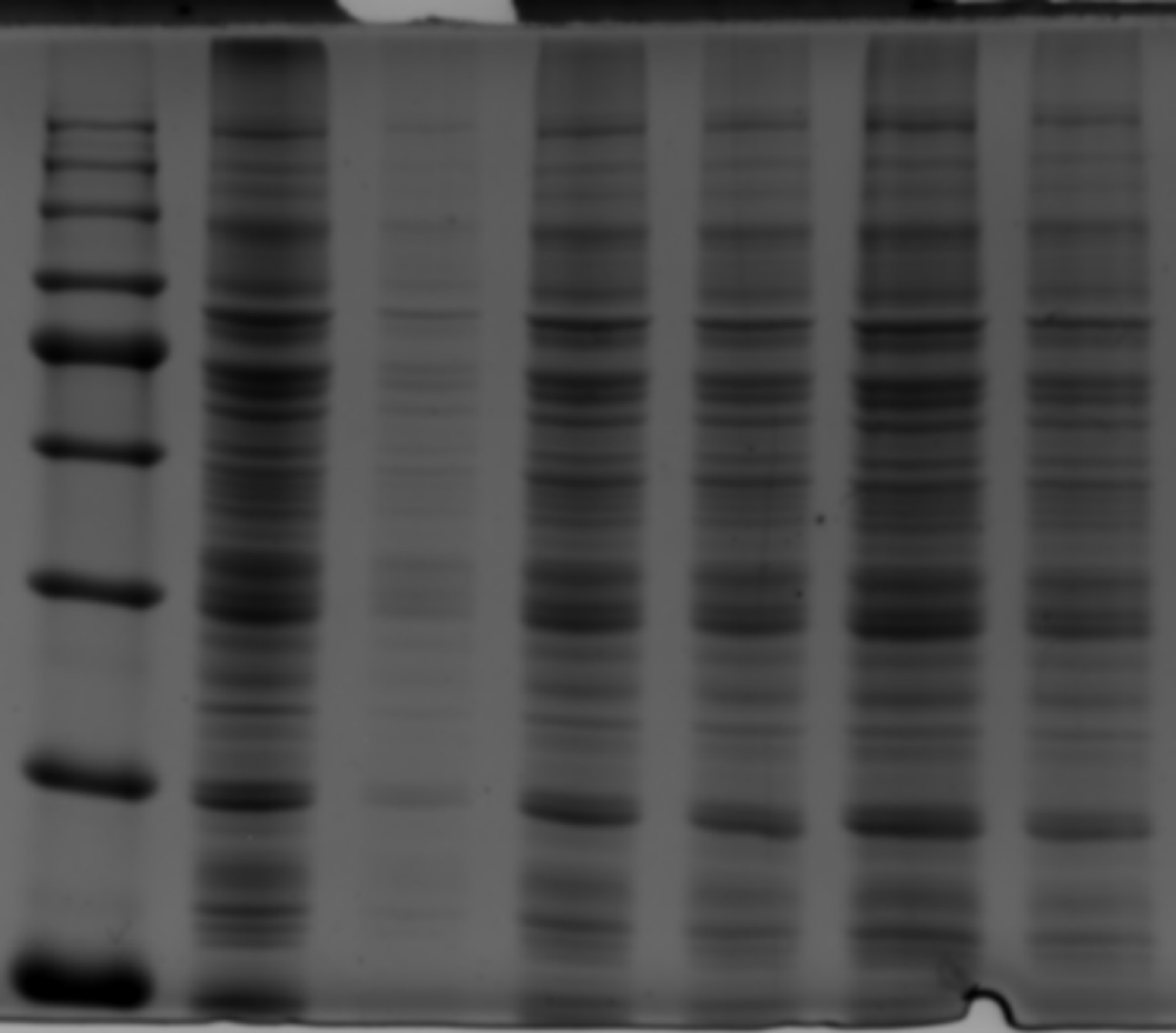


**Figure S2: Whole SDS Page gel user for western blotting. Excerpt marked in red corresponds to expected ku70p band size.** No editing was performed in this image. Lane 1: Protein molecular weight ladder. Lanes 2 and 3: Loading control. Lane 4: 50 µg Negative control (no riboswitch) and no tetracycline. Lane 5: 50 µg Negative control (no riboswitch) 250µM tetracycline. Lane 6: 50 µg TRS ku70 no tetracycline. Lane 7: 50 µg TRS ku70 250 µM tetracycline.


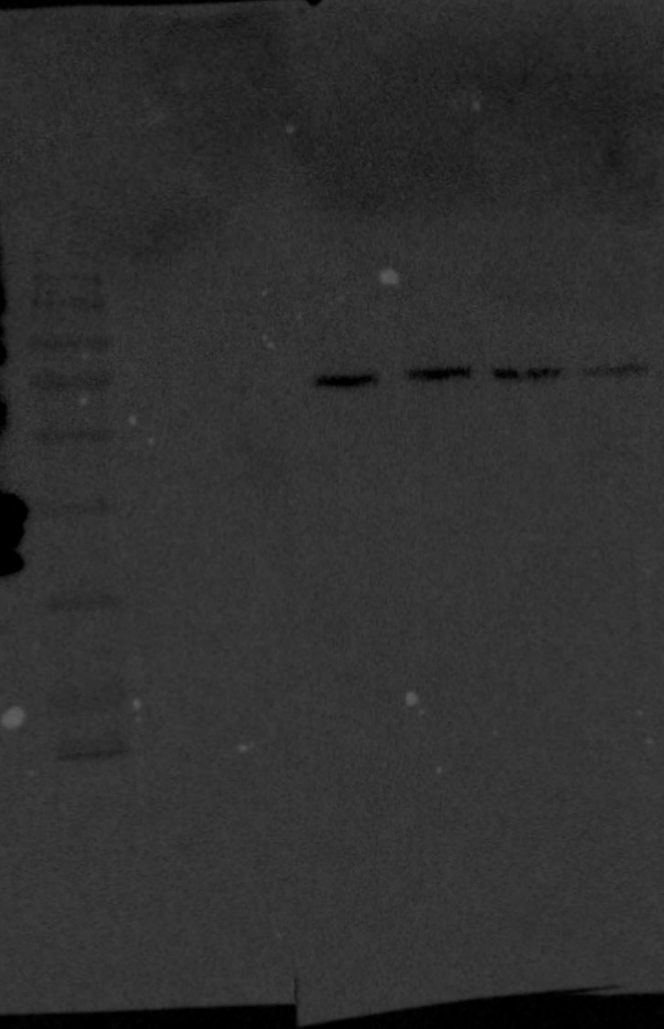


**Figure S3: Whole image of western blotting membrane used in figure 5.** No editing was performed in this image. Lane 1: Protein molecular weight ladder. Lanes 2 and 3: Loading control. Lane 4: 50 µg Negative control (no riboswitch) and no tetracycline. Lane 5: 50 µg Negative control (no riboswitch) 250µM tetracycline. Lane 6: 50 µg TRS ku70 no tetracycline. Lane 7: 50 µg TRS ku70 250 µM tetracycline.
